# Characterization of Antimicrobial Resistance in *Campylobacter* Species from Broiler Chicken Litter

**DOI:** 10.3390/antibiotics14080759

**Published:** 2025-07-28

**Authors:** Tam T. Tran, Sylvia Checkley, Niamh Caffrey, Chunu Mainali, Sheryl Gow, Agnes Agunos, Karen Liljebjelke

**Affiliations:** 1 Department of Ecosystem and Public Health, Faculty of Veterinary Medicine, University of Calgary, 3280 Hospital Dr. NW, Calgary, AB T2N 4Z6, Canada; slcheckl@ucalgary.ca (S.C.); niamh.caffrey@ucalgary.ca (N.C.); kliljebj@ucalgary.ca (K.L.); 2 Alberta Agriculture and Irrigation, 116 Street, Edmonton, AB T6H 4P2, Canada; chunu.mainali@gov.ab.ca; 3 Western College of Veterinary Medicine, University of Saskatchewan, 52 Campus Dr, Saskatoon, SK S7N 5B4, Canada; sheryl.gow@usask.ca; 4 Public Health Agency of Canada, Center for Foodborne, Environmental and Zoonotic Infectious Diseases, 370 Speedvale Avenue West, Suite #201, Guelph, ON N1H 7M7, Canada; agnes.agunos@canada.ca

**Keywords:** *Campylobacter* spp., antibiotic resistance, poultry, conjugation, pathogen

## Abstract

**Background/Objectives:** Campylobacteriosis in human populations is an ongoing issue in both developed and developing countries. Poultry production is recognized as a reservoir for antimicrobial resistance and main source of human *Campylobacter* infection. **Methods**: In this study, sixty-five *Campylobacter* isolates were cultured from fecal samples collected from 17 flocks of broiler chickens in Alberta, Canada over two years (2015–2016). Susceptibility assays and PCR assays were performed to characterize resistance phenotypes and resistance genes. Conjugation assays were used to examine the mobility of AMR phenotypes. **Results**: *Campylobacter jejuni* was the predominant species recovered during both years of sampling. There were no *Campylobacter coli* isolates found in 2015; however, approximately 33% (8/24) of isolates collected in 2016 were *Campylobacter coli*. The two most frequent antimicrobial resistance patterns in *C. jejuni* collected in 2015 were tetracycline (39%) and azithromycin/clindamycin/erythromycin/telithromycin resistance (29%). One isolate collected in 2015 has resistance pattern ciprofloxacin/nalidixic acid/tetracycline. The *tetO* gene was detected in all tetracycline resistant isolates from 2015. The *cmeB* gene was detected in all species isolates with resistance to azithromycin/clindamycin/erythromycin/telithromycin, and from two isolates with tetracycline resistance. Alignment of the nucleotide sequences of the *cmeB* gene from *C. jejuni* isolates with different resistance patterns revealed several single nucleotide polymorphisms. A variety of multi-drug resistance patterns were observed through conjugation experiments. **Conclusions**: These data suggest that poultry production may serve as a potential reservoir for and source of transmission of multi-drug resistant *Campylobacter jejuni* and supports the need for continued surveillance.

## 1. Introduction

*Campylobacter* spp. are Gram-negative bacteria varying in morphology from rod-, comma- or s-shape, having a single polar flagellum, bipolar flagella, or no flagellum [1]. Most species require microaerophilic conditions (5% O_2_, 10% CO_2_, 85% N_2_) for optimal growth. Some *Campylobacter* spp. can grow either in microaerophilic or anaerobic conditions [1]. Some species, such as *Campylobacter concisus*, *Campylobacter curvus*, *Campylobacter rectus*, *Campylobacter mucosalis*, *Campylobacter showae*, *Campylobacter gracilis*, and to a certain extent, *Campylobacter hyointestinalis*, require the presence of hydrogen or formate in culture media.

*Campylobacter* species, especially *C. jejuni* and *C. coli* are the most common cause of diarrheal illness in humans [2,3]. The number of campylobacteriosis cases reported in people in Canada was roughly ten thousand each year during 2006–2015, a rate of approximately 27 cases per 100,000 population [4]. In Europe, there was an estimate of 230,000 cases annually [5], while globally approximately 96 million cases was estimated annually [5]. *C. jejuni* appears to cause 95% of diagnosed campylobacteriosis cases in people [6]. Most *Campylobacter* infections are mild and self-limiting with symptoms of acute watery or bloody diarrhea, fever, abdominal cramps and weight loss [1]. However, infections may become severe and prolonged especially in patients whose immune systems are compromised [7].

*Campylobacter* spp. is transmitted to human from various sources such as untreated drinking water, contaminated meat products or direct contact with live animals [8]. Poultry production is recognized as a reservoir for antimicrobial resistance and main source of human *Campylobacter* infection, especially infection caused by *C. jejuni* [9,10]. *C. jejuni* is a common commensal species in chicken gut microbiome [9]. Broiler meat caused 20–30% of human infections while 50–80% was presumably from chicken reservoir as a whole [8]. Chicken litter is known to be a reservoir for multidrug resistant bacteria due to the misuse of antimicrobials in poultry production [10,11]. In poultry Alberta, 23.5% (48/204) of chicken samples and 14.2% (8/110) of turkey samples were positive with *Campylobacter spp.,* most of which showed resistance to tetracycline [12]. Pig production is also another *Campylobacter* reservoir and mainly associated with *C. coli* [13].

Erythromycin (macrolide) is the drug of choice for *C. jejuni* campylobacteriosis in people because of high effectiveness, low toxicity and ease of administration. Fluoroquinolones (e.g., ciprofloxacin) are the second choice for treatment because of broad spectrum activity [14]. Alternative antibiotic choices such as chloramphenicol, clindamycin, aminoglycosides and carbapenems are effective against *Campylobacter* spp. Despite effective treatments the prevalence of resistant strains may complicate empirical treatment of campylobacteriosis [15]. All antimicrobials used to treat campylobacteriosis are considered to be of very high importance (Category I) or high importance (Category II) for human medicine [16]. The use of Category I and II antimicrobials is now prohibited in poultry for preventive treatment [17].

Horizontal gene transfer (HGT) is considered as a key mechanism for not only driving genetic diversification but also facilitating the dissemination of antimicrobial resistance (AMR) genes. There are three HGT routes: natural transformation, conjugation and bacteriophage transduction, which were previously described, specifically in *Campylobacter* spp. [18]. HGT via conjugative plasmids is the most well-studied mechanism for the spread of AMR genes in resistant bacteria [18,19,20]. The most prevalent plasmid found in both *C. jejuni* and *C. coli* was type-1 plasmid (pTet) harboring *tetO* gene [18]. However, HGT via natural transformation appears to be more efficient for genetic exchange in *Campylobacter* spp. The CmeABC efflux pump in *C. jejuni* comprises a periplasmic fusion protein (CmeA), an inner membrane efflux transporter (CmeB), and an outer membrane protein (CmeC) [21,22]. This predominant efflux pump was shown to actively expel multiple antimicrobials such as fluoroquinolones, macrolides, and tetracycline [21,23]. A recent study demonstrated mutations in RE-CmeB (CmeB in a functionally potent variant of CmeABC), but not in RE-CmeA or RE-CmeC, were responsible for the functional gain of the multidrug efflux pump [24].

In this retrospective study, antimicrobial resistance phenotypes and genotypes of *Campylobacter* strains isolated from commercial broiler chicken production in Alberta during the period of 2015–2016 were characterized. Our study also performed conjugation assays to show the mobility of AMR phenotypes among *Campylobacter* spp. strains.

## 2. Materials and Methods

### 2.1. Flock Characteristics

Management practices, including methods used to clean barn and water lines, and antimicrobials used in flocks were collected and summarized (Table S1). The most commonly used antibiotics were bacitracin (*n* = 14), salinomycin (*n* = 9) and monensin (*n* = 5).

### 2.2. Bacterial Isolation and Speciation

Four pooled fecal samples, representing one per floor quadrant with at least 10 fecal droppings were collected from randomly selected barns and floors (if multiple level/pen barn) in 2015 and 2016, as part of the Canadian Integrated Program for Antimicrobial Resistance Surveillance program–CIPARS [25]. Using a sterile sponge, 2 environmental barn surface samples and 3 meconium samples were collected. In total, 68 fecal samples per year were collected from 17 flocks of broiler chickens in Alberta, Canada (Table S1). Pooled fecal samples were sent on ice in coolers to Alberta Agriculture and Forestry for bacterial isolation and speciation on the same day.

*Campylobacter* was isolated using the standard CIPARS methodology, which is as follows: 25 g portion of each composite fecal sample was mixed with 225 mL of buffered peptone water (BPW) and incubated at 35 ± 1 °C for 24 h. The BPW mixture was serially diluted with Bolton broth (BB) in the ratios of 1:100 and 1:1000, then incubated in a microaerophilic atmosphere at 37 °C for 4 h. After that, they were incubated at 42 °C for 20 to 24 h. The BB tube contents were next streaked on modified Charcoal Cefoperazone Deoxycholate Agar (mCCDA) plates, followed by microaerophilic incubation at 42 °C for 72 h. Finally, presumptive *Campylobacter* colonies were identified using biochemical tests (Gram stain, catalase test, oxidase test) and multiplex PCR for speciation [25].

The multiplex PCR speciation of *C. jejuni* and *C. coli* was performed as previously described [26]. Three pairs of oligo primers were added into the PCR mix (Table 1).

The bacterial isolates were then shipped to University of Calgary for further characterization. Mueller-Hinton agar/broth (MHA/MHB) (Becton Dickinson-BD, Franklin Lakes, NJ, USA) was used to recover these isolates. *Campylobacter* was cultured under microaerophilic condition at 37 °C for 3 days or at 42 °C for 2 days. Microaerophilic conditions were achieved by placing activated sachets in a BD BBL GasPak^TM^ jar (BD GasPak^TM^ EZ Campy Container System, BD, Franklin Lakes, NJ, USA) with inoculated growth medium.

### 2.3. Antimicrobial Susceptibility Assays

The minimal inhibitory concentration (MIC) values for *Campylobacter* were determined by broth microdilution assay as part of the CIPARS program and was performed by Public Health Agency Canada (PHAC). The detailed procedure was previously described in a previous report by PHAC [25]. Briefly, the CAMPY plates designed by NARMS and containing 9 dehydrated antimicrobials were used. The list of these antimicrobials was presented in Table 2 with the testing concentration range for each antimicrobial indicated in clear areas. After incubation period, plates were read using the Sensititre Vizion System. The MIC values obtained were compared with those of CLSI standards [30]. Isolates resistant to at least three drug classes were considered as multiclass resistant (MDR) isolates.

The MIC values for transconjugants (see protocol for transconjugants selection) were also determined using a broth microdilution method. Sensititre™ Campylobacter MIC plates (Trek Diagnostic Systems, Thermo Fisher Scientific Inc., Mississauga, ON, Canada). Strains were streaked on Mueller-Hinton agar plates and incubated in microaerophilic conditions at 37 °C for 72 h or 42 °C for 48 h. Several colonies were selected and inoculated into 5 mL Sensititre™ cation-adjusted Mueller-Hinton broth with TES buffer–CAMHBT (Trek Diagnostic Systems, Thermo Fisher Scientific Inc., Mississauga, ON, Canada) and adjusted to a 0.5 McFarland Standard using a Sensititre™ nephelometer. The inoculated CAMHBT was then mixed well, and subsequently 100 µL was transferred into Sensititre cation adjusted Mueller-Hinton broth with TES buffer and lysed horse blood–CAMHBT+ LHB (Trek Diagnostic Systems, Thermo Fisher Scientific Inc., Mississauga, ON, Canada). The inoculated CAMHBT + LHB was mixed, and 100 µL was inoculated into each well on MIC plate using Sensititre™ Auto-Inoculator. The microtiter plate was incubated in microaerophilic conditions at 37 °C for 48 h before reading results using the Sensititre™ Manual Viewer. For interpretation of manually read results we followed MIC Interpretive guidelines as provided by the CLSI [30].

### 2.4. Genomic DNA Extraction

Genomic DNA extraction was performed using a previously published method with modifications [31]. Briefly, overnight culture was harvested, re-suspended in 500 μL TES (10 mM Tris, 25 mM EDTA, pH 8.0) and lysed using lysis solution (20 μL of 25% SDS, 50 μL of 5 mg/mL of predigested Pronase E and 50 μL of 5 M NaCl) at 68 °C for 30 min. Proteins were precipitated by adding 260 μL of 7.5 M ammonium acetate to the lysate kept on ice for 20 min. Precipitated protein was separated from the lysate by centrifuging at 13,000 rpm for 15 min. DNA was extracted from the lysate supernatant by adding chloroform of the same volume and subsequent centrifugation at 13,000 rpm for 15 min. After this centrifugation step, the top layer was transferred to a new tube containing 780 μL of isopropanol and the mixture was incubated on ice for 30 min to precipitate DNA. DNA was pelleted by being centrifuged at 13,000 rpm for 1 min, then washed with 500 μL of 70% ethanol and pelleted again. After the supernatant was discarded, the tube was air-dried to remove traces of ethanol and then DNA was dissolved in 50 μL of TE buffer.

### 2.5. PCR Assay to Detect AMR Genes

A PCR assay was performed on genomic DNA prep to determine the presence of *tetA*, *tetO* and *cmeB* genes with previously published primers (Table 1).

Two *tetO* PCR products from isolates 96.3 and 13.3 were sent for Sanger sequencing (https://cumming.ucalgary.ca/research/cat/health-genomics, accessed on 13 April 2025) to confirm their sequences. Four *cmeB* PCR products from isolates 13.3, 14.3 85.3 and 86.3 were also sent for Sanger sequencing (https://cumming.ucalgary.ca/research/cat/health-genomics, accessed on 13 April 2025) to confirm their sequences. Multiple sequence alignment was done using online tool (http://multalin.toulouse.inra.fr/multalin/, accessed on 13 April 2025).

### 2.6. Conjugation Assay

All *C. jejuni* isolates displaying various resistance patterns selected for conjugation assays were from the 2015 sampling. The *C. jejuni* isolate number 13.3, with MDR phenotype: azithromycin/clindamycin/erythromycin/telithromycin (AzClErTl) was used for mating assays with four randomly picked *C. jejuni* isolates (6.3, 33.3, 85.3, 96.3) that displayed resistance to tetracycline (Te) but susceptible to macrolides. The donor and recipient strains were mated in the ratio of 1:1 on MHA plates [32]. After 3-day incubation at 37 °C, conjugation spots were transferred to selective media: MHA plates supplemented with erythromycin (5 µg/mL) and tetracycline (5 µg/mL), to select for transconjugants. Conjugation spots were also spotted individually onto MHA plates and then transferred to selective media (MHA plates supplemented with erythromycin (5 µg/mL) and tetracycline (5 µg/mL)), as negative controls.

In a second conjugation experiment, the *C. jejuni* isolate number 96.3 which had a ciprofloxacin/nalidixic acid/tetracycline resistance phenotype (CiNaTe) was used as the donor in a mating assay with three isolates (no. 13.3, 113.3, 117.3) which had the AzClErTl resistance phenotype. The conjugation protocol was similar to the protocol mentioned above, except for the use of different selective media. In this conjugation experiment, the selective medium was MHA supplemented with erythromycin (5 µg/mL) and nalidixic acid (15 µg/mL).

A third conjugation experiment was performed similarly as described in the second conjugation using the same donors and recipients, except for selective media. In the third conjugation, selective media were MHA supplemented with erythromycin (5 µg/mL), tetracycline (5 µg/mL) and nalidixic acid (15 µg/mL).

### 2.7. Statistical Analyses

All analysis was completed in Stata 15 (StataCorp LLC: College Station, TX, USA).

Fisher’s exact test was used to assess resistance to different antimicrobials between 2015 and 2016.

## 3. Results

### 3.1. Distribution Frequency and Minimum Inhibition Concentration (MIC) for Campylobacter *spp.* Isolates from 2015 and 2016

Resistance to one or more drugs was detected in 9/17 flocks. Two flocks had isolates that were either susceptible or intermediate to all drugs, and isolates from the remaining six flocks were susceptible to all drugs tested. Chicks originated from three different hatcheries; however, 80% were from the same hatchery. Two flocks reported no use of antibiotics, the remaining 15 flocks were conventionally raised (i.e., reported using antibiotics).

**Figure 1:**
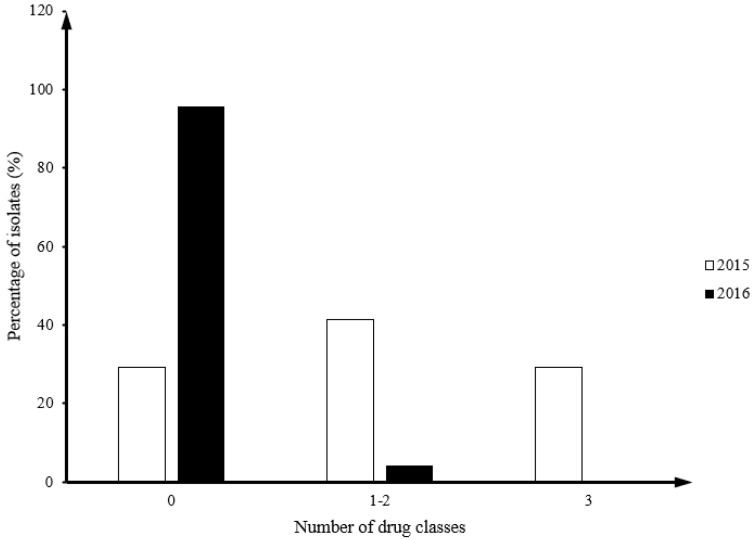
Comparison of distribution frequency by the number of drug classes between *Campylobacter* in year 2015 and 2016.

Forty-one and twenty-four *Campylobacter* isolates were isolated from fecal samples in 17 flocks in 2015 and 2016, respectively. All 2015 isolates (100%, 41/41) were *C. jejuni*, while in 2016 there were 66.7% *C. jejuni* (16/24) and 33.3% *C. coli* (8/24). Twenty nine percent of *Campylobacter* isolates from 2015 (12/41) had MDR isolates, but no MDR isolates were identified in 2016 (Figure 1).

The distribution of MICs around the resistance breakpoint for each antimicrobial showed that some isolates in 2015 had MICs larger than the maximum value of the tested range (Table 2): TEL (12/12), NAL (1/41), ERY (12/41), AZM (12/41). All 2016 isolates had MIC of all antimicrobials falling within the tested range (Table 2).

The association between resistance to individual drugs and year of isolation was examined in *C. jejuni* using Fisher’s exact test. A significant difference in the number of resistant isolates from 2015 (17/41, 41%) to 2016 (1/24, 4%) was detected for tetracycline (*p* = 0.011), and the pattern of telithromycin, erythromycin, clindamycin and azithromycin resistance (*p* = 0.013).

### 3.2. Resistance Patterns of Campylobacter Isolates from 2015 and 2016

Two main resistance patterns found in *Campylobacter* isolates from 2015 were azithromycin/clindamycin/erythromycin/telithromycin resistance (AzClErTl^R^–pattern 1, drug classes: macrolide-lincosamide-ketolide) and tetracycline resistance (Te^R^–pattern 2, drug classes: tetracyclines). In 2015, 39% (16/41) exhibited Te^R^ pattern, and 29% (12/41) exhibited the AzClErTl^R^ pattern. There was only one isolate 96.3 which displayed resistance to ciprofloxacin/nalidixic acid/tetracycline resistance (CiNaTe^R^, drug classes: quinolone-tetracycline).

*C. jejuni* isolates with the resistance pattern AzClErTl^R^ were identified in three flocks in 2015. All three flocks obtained their chicks from the same hatchery, and all three farms used salinomycin for treatment of coccidiosis. These farms had the same floor space area (8000 ft^2^) and stocking density (0.54 ft^2^ per bird), used Ross 308 birds, and were all multi-age facilities [25]. Sanitation on these three farms was done using a hot water wash between productions periods, with chlorine in a pressurized form used as the disinfectant of choice. These three farms did not disinfect their water lines between flocks but did use chlorine to treat the water lines during the production cycle [25].

Out of 16 *C. jejuni* isolates collected in 2016, there was only one isolate which had resistance to Tetracycline (Te^R^). The rest 15 *C. jejuni* and 8 *C. coli* isolates in 2016 were pan-sensitive.

### 3.3. Detection of AMR Genes

All Te^R^ isolates from the 2015 batch (*n* = 17) harbored *tetO* gene as determined by PCR assay and sequence analysis. The *tetO* nucleotide sequence had 99% agreement with the sequence encoding TetM/TetW/TetO/TetS family tetracycline resistance protein published in the GenBank database (Accession No. CP023546.1). The *tetA* gene sequence was not detected in any of our Te resistant isolates. For the PCR assays, *C. jejuni* isolate 13.3 with the AzClErTl^R^ pattern was included as a negative control as no *tetO* gene was amplified from this tetracycline sensitive isolate.

**Figure 2:**
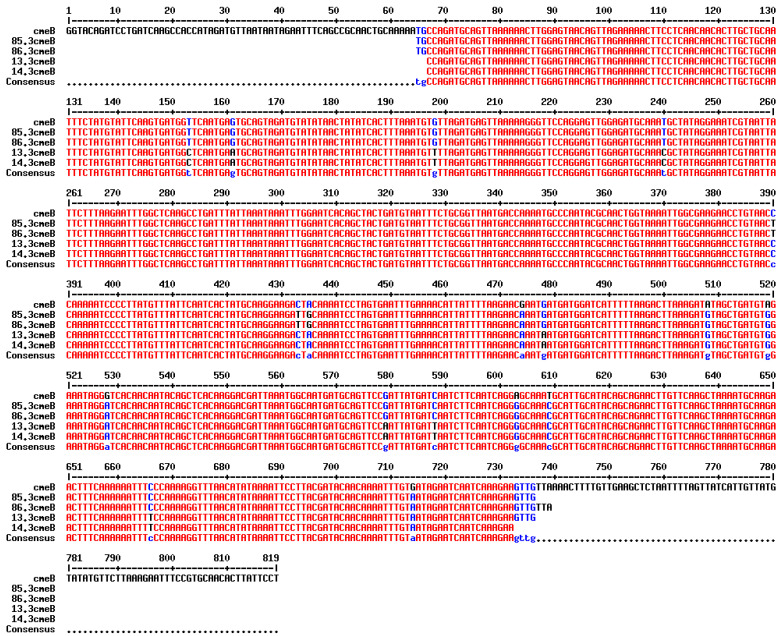
Multiple sequence alignment of *cmeB* gene amplification from four isolates against *cmeB* gene from Genbank database (Access No. AB894099.1) using online tool (http://multalin.toulouse.inra.fr/multalin/, accessed on 13 April 2025). Two of them (85.3 and 86.3) were *C.jejuni* isolates with Te resistance pattern, and the other two (13.3 and 14.3) were *C. jejuni* isolates with AzClErTl resistance pattern.

The *cmeB* gene was detected in all *C. jejuni* strains collected in 2015 which had the AzClErTl^R^ pattern (*n* = 12). In addition, out of five *C. jejuni* isolates which had other resistance patterns (Te^R^ and CiNaTe^R^), the *cmeB* gene was detected in two Te^R^ *C. jejuni* isolates. Alignment of the *cmeB* nucleotide sequence obtained by PCR revealed that isolates with the same resistance pattern (AzClErTl^R^ or Te^R^) shared the same nucleotide sequence and single-nucleotide polymorphisms of the *cmeB* gene (Figure 2). The *cmeB* gene was not detected in a *C. jejuni* isolate with the CiNaTe^R^ pattern nor in two *C. jejuni* isolates with Te^R^ pattern.

### 3.4. Campylobacter jejuni Antimicrobial Resistance Phenotypes Transferred via In Vitro Conjugation

Three out of four *C. jejuni* isolates from 2015 with either Te^R^ (isolates 6.3 and 85.3) or CiNaTe^R^ (isolate 96.3) were able to produce transconjugants when mated with the *C. jejuni* isolate from 2015 with AzClErTl^R^ pattern (isolate 13.3) (Table 3). The combined AMR pattern (AzClErTlTe^R^) of transconjugants 13.6, 13.85 and 13.96 was then confirmed by antimicrobial susceptibility assay (Table 3). All the transconjugants had the same MICs for nalidixic acid as the parental isolate 13.3 (MIC = 8 µg/mL). However, these MICs were higher than MICs in two isolates 6.3 and 85.3 (MIC ≤ 4 µg/mL), but lower than the MIC of the isolate 96.3 (MIC > 64 µg/mL). As a result, the Te^R^ phenotype was likely transferred from supposed donors (6.3, 85.3, 96.3) to a supposed recipient (13.3). Although all of the transconjugants had phenotypic resistance to clindamycin, their MICs (MIC = 8 µg/mL) were half of the supposed recipient’s original MIC value (MIC = 16 µg/mL).

In the second conjugation assay, three isolates (13.3, 113.3 and 117.3) with AzClErTl^R^ pattern were mated with the isolate 96.3 with CiNaTe^R^ pattern (Table 3). All transconjugants displayed the combined patterns CiNa^R^ + AzClErTl^R^ but they were all susceptible to Te; and two of them had an increased MIC (16 µg/mL) for ciprofloxacin as compared to the parental strain (8 µg/mL).

In the third conjugation, the presence of tetracycline in the selective media helped maintain Te resistance in the transconjugants. Presumable *C. jejuni* donors (13.3, 113.3 and 117.3) with AzClErTl^R^ pattern was mated with the presumable *C. jejuni* recipient 96.3 with CiNaTe^R^ pattern and selected on medium containing tetracycline. The transconjugants had a combined multi-drug resistance pattern of CiNaTeAzClErTl (Fluoroquinolones–CiNa, Tetracycline–Te, Macrolides–AzEr, Lincosamide–Cl, Ketolide–Tl).

## 4. Discussion

*Campylobacter jejuni* was the only *Campylobacter* species identified in samples from broiler production in Alberta in 2015, while both *C. jejuni* (66.7%) and *C. coli* (33.3%) were identified in samples collected in 2016. Overall, *C. jejuni* was the predominant *Campylobacter* species isolated, which is similar to other studies [5,33]. This is a clinically grave concern in humans when considering the fact that the majority (95 to 98%) of human cases of *Campylobacter* gastroenteritis were caused by *C. jejuni*, followed by *C. coli* cases (2 to 5%) [2,34]. Our study showed almost 30% of *C. jejuni* isolates from poultry in 2015 having phenotypic resistance to erythromycin. In addition, while other studies otherwise reported that *C. coli* was more likely to be associated with macrolide resistance, macrolide resistance phenotype was only found in *C. jejuni* isolates in our study [35]. All *C. jejuni* isolates were erythromycin-sensitive, while 9% of *C. coli* were resistant to erythromycin from Irish broiler neck skin and caeca [36].

Tetracycline-resistant isolates were found to make up 41% of 2015 Campylobacter isolates. The *tetO* gene was identified in all our 2015 C. jejuni isolates (*n* = 17) with phenotypic resistance to tetracycline. This result is similar to previous studies where the *tetO* gene was identified as the most common tetracycline resistance gene in all *C. jejuni* and *C. coli* isolates with resistance to tetracycline [27,37,38]. Transmissible plasmids carrying the *tetO* gene have been found not only in *C. jejuni* and *C. coli* but also in other bacteria such *Enterococcus faecalis* and *Streptococcus* spp. [39]. These *tetO*-carrying conjugative plasmids were also associated with genes encoding for different aminoglycoside inactivating enzymes, transposase- like genes, and multiple other genes [40,41]. The *tetO* gene can also be located on the bacterial chromosome, especially in *C. coli* [37,38]. The phenotypes of our transconjugants imply that the *tetO* gene in some of our isolates is plasmid-encoded and transmissible. The isolate that was unable to transfer the tetracycline resistance phenotype to the recipient might carry a chromosomally encoded *tetO* gene, or the gene may be located on a separate mobile element which was not transferred.

The cmeB gene encodes for an inner membrane efflux transporter and is a part of three-gene operon (designated cmeABC) that contributes to multidrug resistance in *C. jejuni* [29]. It was shown that mutation of the *cmeB* gene resulted in decreasing MICs to a wide range of antimicrobial agents (i.e., ciprofloxacin, nalidixic acid, erythromycin, tetracycline), heavy metals and bile salts between 2 and 4000-fold. In these *cmeB* mutant strains resistance to ciprofloxacin was decreased 8-fold, resistance to nalidixic acid decreased 2-fold, to erythromycin decreased 4-fold, and tetracycline resistance decreased 8-fold, [29]. In our study, the *cmeB* gene (819 bp) was selected for screening in a subset of *Campylobacter* isolates, especially the ones with AzClErTl phenotype because the gene was likely to confer resistance to antibiotics of different classes. All isolates with AzClErTl resistance pattern carried the *cmeB* gene. The *cmeB* gene was also present in two *C. jejuni* isolates with Te resistance pattern. However, they did not share the same sequence identity of *cmeB* genes with *C. jejuni* isolates of AzClErTl resistance pattern. A significant increase of *cmeB* mutations in *C. jejuni* strains carrying *cmeB* gene compared to those in the CmeB null mutant strains at 10× and 32× the concentration [42]. However, there were no further investigation of these mutations in their study.

In this study, only one *C. jejuni* isolate was resistant to ciprofloxacin, nalidixic acid and tetracycline. The *cmeB* gene was not detected in the *C. jejuni* isolate with the CiNaTe phenotype although this gene was shown to be associated with the resistance to ciprofloxacin, nalidixic acid and tetracycline in several previous studies [29,42,43] We postulate that some mutation in the *gyrA* gene most likely accounted for the resistance to flouroquinolones (ciprofloxacin and nalixidic acid) in this isolate because these drugs target the DNA gyrase encoded by *gyrA* gene [44,45].

In the first conjugation assay, the transconjugants with a combined resistance pattern (AzClErTl + Te) can be made by mating isolates of AzClErTl and Te phenotypes. Based on the MICs of transconjugants, isolates with Te phenotypes were likely to be the donors which transferred Te phenotype into the recipient isolate 13.3 with AzClErTl phenotype. In the second conjugation assay, isolate 96.3 with CiNaTe phenotype was used as the recipient and mated it with isolates exhibiting AzClErTl resistance pattern to determine the transferability of AzClErTl phenotype. Interestingly, the transconjugants displayed resistance not only to erythromycin, which was used as a selection marker, but also to azithromycin, clindamycin, and telithromycin. More surprisingly, all transconjugants maintained resistance to CiNa but lost resistance to Te after conjugation. It is well-known that the strains normally suffer a fitness cost to maintain plasmids; therefore, they can easily lose plasmids in antibiotic-free environment [46]. In the last conjugation experiment, we wanted to see if the transconjugants still maintain resistance to Te when we added tetracycline into the media in addition to nalidixic acid and erythromycin. The results suggested that they were able to maintain resistance to all antimicrobials provided that selection pressure was present in the media.

In conclusion, the study isolated and speciated *Campylobacter* isolates from broiler chickens in Alberta. In both years, *C. jejuni* was the predominant species isolated, and *C. coli* was only isolated in 2016. Twenty nine percent of *C. jejuni* isolates from 2015 (12/41) had multiclass drug resistance (MDR) (≥3 drug classes), but no MDR isolates were identified in 2016. Several single nucleotide polymorphisms were found in the *cmeB* gene of isolates of different resistance patterns. We also showed the potential for resistance pattern transfer during conjugation. The demonstration of transmission of multi-drug resistance via conjugation between strains supports the importance of continued antimicrobial resistance surveillance in food borne pathogens.

## Figures and Tables

**Table 1:** Primers used in the study.

Gene Target (Size)	Primers	Reference
16S universal ribosomal RNA sequence (1062 bp)	16s-F: 5′-GGAGGCAGCAGTAGGGAATA-3′ 16s-R: 5′-TGACGGGCGGTGAGTACAAG-3′	[26]
Aspartokinase to detect *C. coli* (500 bp)	CC18-F: 5′- GGTATGATTTCTACAAGCGAG-3′ CC519-R: 5′-ATAAAAGACTATCGTCGCGTG-3′	[26]
Hippuricase to detect *C. jejuni*	hipO-F: 5′-GACTTCGTGCAGATATGGATGCTT-3′ hipO-R: 5′-GCTATAACTATCCGAAGAAGCCATCA-3′	[26]
*tetA* gene (888 bp)	tetA_F: 5′-GTGAAACCCAACATACCCC-3′ tetA_R: 5′-GAAGGCAAGCAGGATGTAG-3′	[27]
*tetO* gene (559 bp)	tetO_F: 5′GGCGTTTTGTTTATGTGCG-3′ tetO_R: 5′-ATGGACAACCCGACAGAAGC-3′	[28]
*cmeB* gene (819 bp)	cmeB_F: 5′-GGTACAGATCCTGATCAAGCC-3′ cmeB_R: 5′-AGGAATAAGTGTTGCACGGAAATT-3′	[29]

**Table 2:** Minimum inhibitory concentration (MIC)’s distributions for resistance to each drug in *Campylobacter* isolates from Alberta poultry farms in year 2015 (*n* = 41) and in year 2016 (*n* = 24).

**Year**	**Antimicrobial**		**Distribution of Isolates (Count) Across MIC Range**													
		<0.015	0.015	0.03	0.06	0.12	0.25	0.5	1	2	4	8	16	32	64	>64
	Gentamicin							1	40							
	Telithromycin							21	8				12			
	Clindamycin				6	22	1					5	7			
	Nalidixic acid										27	13				1
2015	Ciprofloxacin					40						1				
	Erythromycin						18	11								12
	Azithromycin	1		14	9	4	1									12
	Florfenicol							3	38							
	Tetracycline				2	5	17							6	2	9
	Gentamicin							6	18							
	Telithromycin							9	7	1	3	4				
	Clindamycin				2	13	2	7								
	Nalidixic acid										7	7	9	1		
2016	Ciprofloxacin				9	15										
	Erythromycin					1	12	3	2	6						
	Azithromycin			13	3	3	5									
	Florfenicol								20	4						
	Tetracycline					14	1				1	7			1	

MIC range from 0.015 to 64 μg/mL were used to test *Campylobacter* isolates. Note: Clear areas indicate the range tested for each antimicrobial. Numbers in shaded areas indicate isolates having MIC outside tested range. Double vertical bars represent resistance breakpoints. Single vertical bars represent susceptibility breakpoints.

**Table 3:** Minimum inhibition concentration (MIC) and AMR patterns of isolates used in conjugation assays and the resulting transconjugants.

Strains	MIC (µg/mL)									Interpretation									AMR Patterns
	AZM	CIP	CLI	ERY	FLR	GEN	NAL	TEL	TET	AZM	CIP	CLI	ERY	FLR	GEN	NAL	TEL	TET	
*C. jejuni* ATCC 33250 ^a^	0.03	0.06	0.25	<0.25	1	0.5	≤4	<0.5	<0.12	S	S	S	S	S	S	S	S	S	Susceptible
6.3	0.12	0.12	0.12	0.5	1	1	≤4	1	**>64**	S	S	S	S	S	S	S	S	**R**	Te
85.3	0.12	0.12	0.12	0.5	1	0.5	≤4	0.5	**>64**	S	S	S	S	S	S	S	S	**R**	Te
96.3	0.06	**8**	0.12	0.25	1	1	**>64**	0.5	**>64**	S	**R**	S	S	S	S	**R**	S	**R**	CiNaTe
13.3	**>64**	0.12	**16**	**>64**	1	1	8	**>8**	0.25	**R**	S	**R**	**R**	S	S	S	**R**	**S**	AzClErTl
113.3	**>64**	0.12	**8**	**>64**	1	1	8	**>8**	0.25	**R**	S	**R**	**R**	S	S	S	**R**	S	AzClErTl
117.3	**>64**	0.12	**16**	**>64**	1	1	8	**>8**	0.25	**R**	S	**R**	**R**	S	S	S	**R**	S	AzClErTl
13.6 ^b^	**>64**	0.12	**8**	**>64**	1	0.25	8	**>8**	**>64**	**R**	S	**R**	**R**	S	S	S	**R**	**R**	AzClErTlTe
13.85 ^b^	**>64**	0.12	**8**	**>64**	1	0.25	8	**8**	**>64**	**R**	S	**R**	**R**	S	S	S	**R**	**R**	AzClErTlTe
13.96 ^b^	**>64**	0.12	**8**	**>64**	2	0.25	8	**>8**	**>64**	**R**	S	**R**	**R**	S	S	S	**R**	**R**	AzClErTlTe
96.13 ^c^	**>64**	**16**	**16**	**>64**	1	0.5	**>64**	**>8**	0.12	**R**	**R**	**R**	**R**	S	S	**R**	**R**	S	CiNaAzClErTl
96.113 ^c^	**>64**	**16**	**16**	**>64**	2	0.5	**>64**	**>8**	0.25	**R**	**R**	**R**	**R**	S	S	**R**	**R**	S	CiNaAzClErTl
96.117 ^c^	**>64**	**8**	**16**	**>64**	1	0.25	**>64**	**>8**	0.12	**R**	**R**	**R**	**R**	S	S	**R**	**R**	S	CiNaAzClErTl

**Note:** Grey shading separates transconjugants from isolates used in conjugation experiments. S: Susceptible, R: Resistant (in bold). AZM/Az = Azithromycin, CIP/Ci = Ciprofloxacin, CLI/Cl = Clindamycin, ERY/Er = Erythromycin, FLR = Florfenicol, GEN = Gentamicin, NAL/Na = Nalidixic acid, TEL/Tl = Telithromycin, TET/Te = Tetracycline. ^a^ Negative control strain was used in antimicrobial susceptibility tests. ^b^ Transconjugants 13.6, 13.85 and 13.96 were obtained via conjugation between the isolate 13.3 with isolates 6.3, 85.3 and 96.3 in the first conjugation assay, respectively. ^c^ Transconjugants 96.13, 96.113 and 96.117 were obtained via conjugation between the isolate 96.3 with isolates 13.3, 113.3 and 117.3 in the second conjugation assay, respectively.

## Data Availability

The original contributions presented in this study are included in the article/Supplementary Materials. Further inquiries can be directed to the corresponding author.

## Supplementary Materials

The following supporting information can be downloaded at: https://www.mdpi.com/article/10.3390/antibiotics14080759/s1, Table S1: Flock level characteristics including antibiotics given in feed and methods used to disinfect barns.
