# Supplementary material for: Characterization of Antimicrobial Resistance in Campylobacter Species from Broiler Chicken Litter
Source: Antibiotics (Basel). 2025 Jul 28;14(8):759. doi: 10.3390/antibiotics14080759 (PMC12383096; doi:10.3390/antibiotics14080759)
Supplement: Supplementary file 1 [file antibiotics-14-00759-s001.zip › antibiotics-3703190-supplementary.pdf]

Characterization of Antimicrobial Resistance in *Campylobacter* Species from Broiler Chicken Litter

Tam T. Tran [1,*,†], Sylvia Checkley [1], Niamh Caffrey [1], Chunu Mainali [2], Sheryl Gow [3], Agnes Agunos [4] and Karen Liljebjelke [1]

[1]Department of Ecosystem and Public Health, Faculty of Veterinary Medicine, University of Calgary, 3280 Hospital Dr. NW, Calgary, AB, Canada T2N 4Z6

[2]Alberta Agriculture and Forestry, 116 Street, Edmonton, AB, Canada T6H 4P2

[3]Western College of Veterinary Medicine, University of Saskatchewan, 52 Campus Dr, Saskatoon, SK, Canada S7N 5B4

[4]Public Health Agency of Canada, Center for Foodborne, Environmental and Zoonotic Infectious Diseases, 370 Speedvale Avenue West, Suite #201, Guelph, ON N1H 7M7

*Corresponding author:

Tam Tran. Current mailing address: Current address: NORCE Research AS, Nygårdstangen, 5838 Bergen, Norway. Email: tran@norceresearch.no.

Table S1. Flock level characteristics including antibiotics given in feed and methods used to disinfect barns

Farm	Isolates	Avilamycin	Bacitracin	Lasalocid	Monensin	Nicarbazin	Narasin	Narasin & Nicarbazin	Salinomycin	Tylosin	Virginiamycin	Penicillin & Streptomycin in	Lincomycin & Spectinomycin at	Disinfect barn with Chlorine	All in all out operation	Use hydrogen peroxide to treat [illegible]	Use chlorine to treat water lines during production
187	85.3-86.3-87.3-88.3		Y		Y								Y	Y	Y		
189	45.3-46.3-47.3-48.3		Y						Y						Y	Y	
317	29.3-30.3-31.3-32.3		Y				Y	Y									Y
318	13.3-14.3-15.3-16.3				Y	Y			Y	Y				Y			Y
323	5.3-6.3-7.3-8.3		Y		Y									Y	Y	Y	Y
368	33.3-34.3-35.3-36.3																
369	37.3-38.3-39.3-40.3																
374	94.3-95.3-96.3		Y					Y	Y				Y	Y	Y	Y	
375	113.3-114.3-115.3-116.3		Y						Y			Y		Y			Y
376	105.3-107.3		Y					Y	Y		Y				Y		Y
378	117.3-118.3-119.3-120.3		Y						Y			Y		Y			Y
432	1-2-3-4		Y		Y										Y	Y	
433	5-6-7-8		Y		Y											Y	
489	65-66-67-68		Y						Y						Y	Y	
491	61-62-63-64		Y						Y							Y	
493	49-50-51-52		Y						Y					Y	Y	Y	
513	101-102-103-104	Y	Y	Y				Y			Y				Y	Y	
